# Nasal Symptoms in Patients with Obstructive Sleep Apnoea and Their Association with Continuous Positive Airway Pressure Usage

**DOI:** 10.3390/life12020305

**Published:** 2022-02-17

**Authors:** Konstantinos Chaidas, Kallirroi Lamprou, Amberley Munnings, John R. Stradling, Annabel H. Nickol

**Affiliations:** 1 Ear, Nose and Throat Department, Oxford University Hospitals NHS Foundation Trust, Oxford OX3 9DU, UK; amberley.munnings@nhs.net; 2 Oxford Centre for Respiratory Medicine, Oxford University Hospitals NHS Foundation Trust, Oxford OX3 7LE, UK; lamproukallirroi@gmail.com (K.L.); john.stradling@ouh.nhs.uk (J.R.S.); annabel.nickol@ouh.nhs.uk (A.H.N.)

**Keywords:** nasal complaints, rhinorrhoea, nasal obstruction, allergic rhinitis, CPAP adherence, CPAP predictors

## Abstract

The role of nasal symptoms in continuous positive airway pressure (CPAP) tolerance is not completely clear. This study aimed to investigate the association between CPAP usage and nasal symptoms, either prior to, or developing during, CPAP use in patients with obstructive sleep apnoea (OSA). Two hundred thirty patients were studied and divided into high-, low-, and non-CPAP users. Nasal symptoms and related quality of life parameters were evaluated prior to CPAP initiation and after three months. We also investigated predictive factors for CPAP usage. Non-CPAP users had significantly worse baseline scores for runny nose compared with high and low users (1.34 vs. 0.68 and 0.75, respectively, *p* = 0.006). There were no other significant differences between the groups. Runny nose was an independent predictive factor for lower CPAP usage (*p* = 0.036). An evaluation after three months showed worsening in runny nose score in high-CPAP users (*p* = 0.025) but not in low- and non-users. There were no significant changes in other nasal symptoms. Our study demonstrates that nasal symptoms were very common in this population but rhinorrhoea was the only symptom associated with poorer CPAP adherence. Moreover, rhinorrhoea worsened after a three-month trial of high-CPAP usage.

## 1. Introduction

Obstructive sleep apnoea (OSA) is a common respiratory disorder affecting 9–38% of the adult population [1] and is associated with daytime sleepiness, impaired cognitive ability, and serious sequelae such as road traffic accidents, cardiovascular morbidity, and all-cause mortality [2]. Continuous positive airway pressure (CPAP) is considered as the gold-standard treatment, especially in moderate and severe cases. CPAP is highly efficacious for the majority of patients, with good long-term adherence up to 80% and 71% at 5 and 10 years, respectively [3]. However, this still leaves a substantial minority of patients poorly tolerant of CPAP.

Many factors, such as OSA severity, have been identified as influencing CPAP usage but much of the variance is left unexplained [3]. Side effects of CPAP are present in 15% to 45% of patients and complaints such as skin irritation, dry mouth, air leak, mask discomfort, claustrophobia, and nasal symptoms have been reported as reasons for poor adherence to CPAP therapy [4]. Nasal symptoms are common in patients with OSA, but their role in OSA and CPAP tolerance is not completely clear [5]. Moreover, the actual effect of CPAP on the nasal cavity is not fully understood. There are conflicting data on the relevance of nasal symptoms, either prior to, or developing during, CPAP usage. This study looks at both these issues by studying a cohort of patients diagnosed with symptomatic OSA and going on to CPAP. We hypothesised that a higher burden of nasal symptoms would be associated with poorer adherence to CPAP, particularly if nasal symptoms worsened following initiation on CPAP.

## 2. Materials and Methods

### 2.1. Study Protocol

Patients aged over 18 years old with a new diagnosis of OSA based on their history and a sleep study, in whom a trial of CPAP was indicated, were recruited consecutively and verbal consent was obtained. Patients were excluded from the study if they were on treatment with systemic or topical medications that might affect nasal symptoms such as oral or nasal corticosteroids, antihistamines, and nasal decongestants.

Prior to CPAP initiation, a full medical history was obtained, and the following parameters were evaluated: age, sex, smoking history, body mass index (BMI), neck circumference, Mallampati grade, co-morbidities, and current medical treatment. The severity of OSA was evaluated based on the oxygen desaturation index (ODI) during an overnight sleep study. Patients were set-up on CPAP, with heated humidification and oronasal mask. Three months after initiating CPAP therapy, CPAP pressure and adherence were noted, and these data were available for all patients through remote review of data from their machine with patient consent.

Patients completed questionnaires at baseline and three months after initiating CPAP therapy where feasible, including the following:

(a) Daytime sleepiness was assessed using the Epworth Sleepiness Scale (ESS) [6].
(b) Nasal breathing was evaluated with a visual analogue scale (VAS), where a score of 0 indicates the absence of nasal obstruction and a score of 10 indicates complete nasal obstruction. A score of 2 or less defines asymptomatic individuals, a score between 2 and 5 mild symptoms, and a score greater than 5 moderate-severe nasal obstruction.
(c) Assessment for the presence of allergic rhinitis was performed via the ‘score for allergic rhinitis’ questionnaire (SFAR) [7]. SFAR is a validated questionnaire aiming to identify patients with allergic rhinitis (AR), which consists of eight questions with a total score ranging between 0 and 16. Individuals with a score of 7 or higher are considered as positive for AR.
(d) Nasal side effects and related quality of life were assessed by using the validated Mini Rhinoconjunctivitis Quality of Life Questionnaire (Mini RQLQ) [8]. The Mini RQLQ consists of 14 items in 5 domains (activity limitations, practical problems, nose symptoms, eye symptoms, and other problems) and each question can be answered on a 7-point scale (0 = not troubled, 6 = extremely troubled).

The whole group was divided into ‘high’, ‘low’, and ‘non’ users based on CPAP adherence and comparisons of various parameters were made. High usage was arbitrarily defined as CPAP use for at least 4 h per night for over 70% of the nights [9], whereas patients using CPAP for less than 4 h per night were considered as low users. Those who discontinued CPAP therapy or declined its use were considered as non-users. We also investigated the presence of predictive factors for CPAP usage. We evaluated changes prior to and after the CPAP trial in each group over a three-month period. Changes between baseline and follow-up in CPAP users with or without allergic rhinitis were also assessed.

### 2.2. Statistical Analysis

#### 2.2.1. Prevalence of Nasal Symptoms and Association with CPAP Adherence

Data are presented as mean ± standard deviation or as a percentage. Continuous and categorical variables were compared using one-way analysis of variance or chi-squared test, as appropriate. One-way analysis of variance was followed by post hoc tests for pair comparisons between groups. Independent predictive factors of CPAP usage were identified by performing multiple linear regression analysis starting with a model of all variables with a *p* < 0.10 associated with their coefficients in the unadjusted univariate analysis. The selection of variables included in the final model was based on the statistical significance of their coefficients.

#### 2.2.2. Changes in Nasal Symptoms after a Three-Month Course of CPAP

Changes in outcome variables before and after CPAP were compared by Wilcoxon signed-rank test. Categorical variables were compared using a chi-squared test. Analysis of changes was performed on high-, low-, and non-CPAP users. The multiple linear regression model was performed again, as above, but now including change in nasal symptoms as a further independent predictor.

*p* values < 0.05 were considered statistically significant. No adjustment was made for multiple comparisons in this exploratory study. All data were statistically analysed using SPSS software for Windows version 19.0 (SPSS Inc., Chicago, IL, USA).

## 3. Results

### 3.1. Prevalence of Nasal Symptoms and Association with CPAP Adherence

Two hundred thirty patients were recruited. Table 1 shows the baseline characteristics of all study participants also divided into high-, low-, and non-CPAP users. One hundred and sixty five of 230 patients (71.7%) continued using CPAP after three months and 109 patients (47.4%) had high CPAP adherence. The percentage of study participants with nasal obstruction prior to CPAP initiation was 57.9%, of whom it was mild in 37% and moderate-severe in 20.9%. Fifty-four patients (23.5%) had an SFAR score indicative of allergic rhinitis. There was no statistically significant difference in most nasal symptoms between the groups, with the exception of runny nose (*p* = 0.006). Specifically, non-CPAP users had significantly worse baseline scores for runny nose compared with high (1.34 vs. 0.68, *p* = 0.006) and low users (1.34 vs. 0.75, *p* = 0.047). There were no other statistically significant differences between the groups apart from CPAP pressure delivered, but this difference is not clinically significant. In our centre, CPAP pressure is determined in part by OSA severity, and the high-CPAP users had numerically more severe OSA, though this was not statistically significant.

Multiple regression analysis confirmed that runny nose was the only independent predictive factor for CPAP usage, with a high baseline score having a negative impact on use (*p* = 0.036; Table 2).

### 3.2. Changes in Nasal Symptoms after a Three-Month Course of CPAP

Of 150 consecutive patients initially recruited, 103 individuals completed the follow-up assessment of nasal symptoms after three months. There was no difference in baseline characteristics in this sub-group compared to the group as a whole. Table 3 shows the changes for high-, low-, and non-users. As expected, symptoms associated with OSA (ESS, sleep, tiredness, and feeling of irritability) improved with a high degree of statistical significance in high-CPAP users. Interestingly, there was an improvement in ESS score in low- and non-CPAP users, as well, although to a less degree. In general, there were no significant changes in nasal symptoms except for an increase in runny nose score in high-CPAP users (*p* = 0.025).

An evaluation of changes after CPAP in high users with and without allergic rhinitis (AR) revealed that non-AR patients (n = 40) had a significant worsening in rhinorrhoea after 3 months (paired difference FU-baseline: 0.55 ± 1.34, *p* = 0.048). Patients with allergic rhinitis (n = 13) also had worse scores for runny nose at follow-up (paired difference FU-baseline: 0.31 ± 2.25, *p* = 0.57), but this did not reach statistical significance, probably due to the low number of patients in the AR group. The VAS scores for nasal obstruction were relatively unchanged in both groups with a paired difference (FU-Baseline) of −0.15 ± 4.14 (*p* = 0.94) in AR patients and −0.05 ± 2.87 (*p* = 0.64) in non-AR patients. When changes in nasal symptoms were included in the linear regression model of predictors of CPAP usage, there were no significant new predictors, apart from a change in tiredness, as would be expected (Table 4).

## 4. Discussion

We have demonstrated that the prevalence of nasal symptoms is high in patients initiating treatment with CPAP for OSA, even once those with pre-diagnosed nasal conditions have been excluded. Almost 58% of patients reported nasal obstruction and 23.5% reported symptoms in keeping with allergic rhinitis. Our hypothesis that nasal symptoms would be a barrier to CPAP use was not borne out by our data for all factors apart from a runny nose, with this being the only symptom where higher baseline symptom burden was associated with subsequent lower hours of CPAP use. We speculate that patients persevere with CPAP because of the benefit they derive in sleepiness and general well-being.

Nasal obstruction secondary to allergic rhinitis or other underlying pathology has been identified as a risk factor for sleep apnoea [10]. Allergic rhinitis is an inflammatory condition of the nasal mucosa affecting 10–30% of the adult population, and is characterised by one or more symptoms, including sneezing, itching, nasal congestion, and rhinorrhoea [11]. Almost one in four (23.5%) study participants suffered from AR even though those with a pre-existing diagnosis of rhinitis had been excluded from this observational study, suggesting that the actual prevalence in the local OSA population is even higher. Shadan et al. found that 37% of OSA patients were affected by allergic rhinitis [12]. There was no difference in AR incidence at baseline between high-, low-, and non-CPAP users in our study.

The use of CPAP affects nasal symptoms, but its effect remains controversial as previous studies have shown mixed results. The presence of positive airway pressure can lead to nasal complaints, such as nasal obstruction, rhinorrhoea, nasal dryness, and sneezing in up to 44–65% of CPAP users [4,13]. Balsalobre et al. showed that CPAP use by awake healthy individuals resulted in worsening of nasal obstruction, which was more evident in those with a known history of AR [14]. Yang et al. showed exacerbation of rhinitis-related symptoms after CPAP within the first year in patients without pre-existing rhinitis and within the second year in known rhinitic patients, suggesting that CPAP therapy may increase the incidence of AR [15].

Interestingly, some studies have revealed improvement of nasal symptoms post-CPAP. Willing et al. demonstrated a decrease in nasal resistance during CPAP therapy in healthy individuals [16]. Cisternas et al. showed a worsening of nasal dryness in non-rhinitic patients, but an improvement of nasal complaints in AR patients [17]. Pitts et al. revealed an improvement in nasal patency in OSA patients using long-term CPAP and even in those with limited CPAP use, with greater improvement in patients with narrower nasal cavities [18].

Our study revealed a significant increase in the score for runny nose after high-CPAP usage, though we acknowledge that there is the possibility that this may have occurred due to chance in view of the large number of observations made. Worsening rhinorrhoea was also noted in low-CPAP users, although this was not statistically significant. In contrast, the score for runny nose remained unchanged in non-users, suggesting that positive airway pressure contributes to rhinorrhoea. Runny nose worsened after CPAP in both AR and non-AR groups, although it did not reach statistical significance in the AR group, probably due to the relatively low number of subjects in this group. On the other hand, there was no significant change in other nasal symptoms including nasal obstruction. Previous studies have shown that CPAP treatment can lead to nasal inflammation which, however, does not necessarily have any clinical implications [17,19]. The addition of humidification may be associated with improved neutrophilic infiltration [20] and a decrease in nasal side effects [21]. It is, thus, likely that the use of CPAP with heated humidification by our study participants has played a role in the prevention of nasal dryness and oedema, although it did not have a favourable effect on rhinorrhoea.

CPAP adherence is variable and has been identified as a limiting factor of this therapeutic modality for patients with OSA. Our study showed that high-CPAP adherence was 47.4%, whereas 71.7% of patients continued using CPAP after a three-month period. This is comparable with previous studies showing a CPAP compliance rate between 30% and 60% [9].

Several factors have been identified as affecting adherence to CPAP including claustrophobia or discomfort caused by the mask, excessive air leak, mouth dryness, and choking sensation [22,23]. Nasal complaints present either prior or secondary to CPAP have also been identified as predictors for CPAP adherence. Others have shown that nasal obstruction is an important factor for CPAP intolerance and increased nasal resistance prior to CPAP initiation is associated with early CPAP discontinuation [24,25]. Li et al. found that CPAP use was significantly lower in patients with smaller nasal passages [26].

Interestingly, our study did not reveal an association between nasal obstruction and CPAP usage. Our findings concur with those published by Skoczynski et al. who observed that acceptance of CPAP therapy was not correlated with nasal patency and Pitts et al. who found that CPAP adherence had no correlation with any measure of nasal patency [18,19]. There was a significant difference in the baseline score for runny nose between CPAP users and non-users and our model shows that rhinorrhoea is an independent predictive factor for low-CPAP usage. There was also significant worsening of rhinorrhoea after high-CPAP usage for three months, but there was no association between changes in nasal symptoms and CPAP adherence. Change in tiredness was the only additional predictor, as would be expected, as it is well demonstrated that CPAP therapy significantly improves daytime sleepiness and tiredness [27].

In most previous studies, a nasal mask was the primary mask utilised, as nasal CPAP is associated with better adherence, lower residual AHI, and higher therapeutic levels compared to oronasal CPAP [28]. However, nasal obstruction and oral breathing are common among patients with OSA and can lead to oral air leak due to mouth opening as a potential adverse effect of CPAP [29]. It has been suggested that patients with oral breathing may be less adherent to nasal CPAP [22]. Patients with nasal pathology and/or nasal symptoms may find it easier to use a full-face mask instead of a nasal mask as the positive air pressure can bypass reduced nasal patency and minimise CPAP-related nasal side effects. All study participants were given an oronasal mask and the delivery route may have influenced our findings and, specifically, the absence of increased nasal complaints after CPAP apart from rhinorrhoea and the lack of association between nasal obstruction and CPAP usage.

The initial adherence to CPAP has been the best predictor for long-term compliance and CPAP usage gradually decreases in patients with nasal complaints [30]. Therefore, it is important to evaluate OSA patients prior to CPAP initiation aiming to treat nasal symptoms and CPAP-related adverse effects such as rhinorrhoea before and during CPAP therapy. Despite not currently being part of our Unit’s standard practice, a clinical assessment by an otorhinolaryngologist prior to CPAP initiation may be beneficial in these patients in order to identify and manage underlying nasal pathology potentially affecting CPAP usage. Humidification, nasal douching, nasal steroids, and nasal surgery are considered the main pillars of the management of nasal symptoms. A systematic review and meta-analysis by Camacho et al. [31] showed that nasal surgery results in a significant overall reduction in CPAP pressures and increase in CPAP adherence with the most effective surgery type being the combination of septoplasty with turbinoplasty. In addition to nasal examination, a full upper airway assessment is necessary in order to exclude other pathologies potentially contributing to upper airway obstruction, breathing difficulties, high CPAP pressure requirements and low-CPAP usage such as tonsillar hypertrophy, tongue base prominence, and/or epiglottic collapse [32].

This study has certain limitations. First, nasal examination was not performed; however, the assessment of nasal symptoms was carried out via validated questionnaires. The SFAR questionnaire was used for the diagnosis of allergic rhinitis without performing radioallergosorbent (RAST) or skin-prick tests. The SFAR score has 74% sensitivity and 83% specificity but is a quick and relatively reliable tool [7]. In order to better evaluate the effect of CPAP on the nasal cavity, certain exclusion criteria were used and for that reason, study participants may differ from the general population. Namely, we excluded patients with known nasal pathology or current medication affecting nasal mucosa and, therefore, the prevalence of nasal symptoms and AR in the general OSA population may be higher than in our study. In contrast with most of the similar studies, we used ODI instead of the apnoea-hypopnoea index (AHI) to make the diagnosis of OSA and classify its severity. The symptoms of OSA are closely related to oxygen desaturations and although AHI is widely used, ODI is as valuable as AHI in diagnosing and grading OSA [33] with a strong correlation of 0.97 with AHI [34]. The follow-up assessment was performed 3 months after initiating CPAP therapy and although this could be considered as a potential weakness, the vast majority of patients demonstrate intolerance to CPAP within the first month of use with early CPAP adherence being reported as the greatest predictor for long-term CPAP adherence [35]. We therefore presume that our results are also related to long-term effects. Likewise, nasal side effects, if any, are expected to be present after three months of CPAP use.

## 5. Conclusions

Despite the high prevalence of nasal symptoms in OSA patients, our study did not demonstrate an association between most nasal symptoms and CPAP usage, apart from rhinorrhoea. Runny nose seems to be an independent predictive factor for poorer CPAP adherence. Moreover, rhinorrhoea worsens after a three-month trial of CPAP, especially in high users. However, given the high frequency of nasal symptoms, it may still be worth sleep practitioners enquiring about their presence and considering treatment. The pathophysiological mechanism generating increased rhinorrhoea in CPAP therapy is not fully understood and further studies to evaluate the effect of humidification and oronasal CPAP masks on the nasal mucosa will be required.

## Figures and Tables

**Table 1:** Characteristics of study population at baseline and comparison between high-, low-, and non-CPAP users.

Variables	Study Participants (n = 230)	High CPAP Users (n = 109)	Low CPAP Users (n = 56)	Non-CPAP Users (n = 65)	*p*-Value
Age, years	51.4 ± 13.1	52.9 ± 11.8	49.2 ± 13.1	50.7 ± 15.1	0.20
Male sex	171 (74.3)	82 (75.2)	43 (76.8)	46 (70.8)	0.72
Smoking					0.58
Non-smokers	194 (84.3)	95 (87.2)	44 (78.6)	55 (84.6)	
Ex-smokers	9 (3.9)	3 (2.8)	4 (7.1)	2 (3.1)	
Smokers	27 (11.7)	11 (10.1)	8 (14.3)	8 (12.3)	
Previous nasal surgery	16	7	5	4	0.80
BMI, kg/m^2^	36.6 ± 8.9	35.9 ± 8.8	36.7 ± 8.4	37.9 ± 9.3	0.34
Neck circumference, inches	17.1 ± 1.7	17.1 ± 1.7	17.3 ± 1.6	17.1 ± 1.9	0.76
Mallampati scale					0.72
Grade 1	33 (14.3)	13 (11.9)	11 (19.6)	9 (13.8)	
Grade 2	104 (45.2)	49 (45.0)	25 (44.6)	30 (46.2)	
Grade 3	87 (37.8)	43 (39.4)	20 (35.7)	24 (36.9)	
Grade 4	6 (2.6)	4 (3.7)	0 (0)	2 (3.1)	
ODI, episodes/hour	40.9 ± 29.2	43.7 ± 32.9	34.9 ± 23.1	41.3 ±27.1	0.19
OSA severity					0.72
Mild	29 (12.6)	13 (11.9)	8 (14.3)	8 (12.3)	
Moderate	74 (32.2)	31 (28.4)	21 (37.5)	22 (33.8)	
Severe	127 (55.2)	65 (59.6)	27 (48.2)	35 (53.8)	
CPAP use per day, hours	3.7 ± 2.8	6.2 ± 1.2	2.8 ± 1.1	0.2 ± 0.2	
CPAP pressure, cm H_2_O	10.5 ± 1.7	10.9 ± 1.9	10.2 ± 1.2	10.1 ± 1.6	0.005 *
Patient questionnaires					
ESS score/24	11.3 ± 5.2	11.3 ± 5.3	11.1 ± 4.4	11.6 ± 5.6	0.88
VAS score/10	3.06 ± 2.93	3.01 ± 2.97	2.84 ± 2.45	3.34 ± 3.27	0.63
Nasal obstruction category					0.29
Asymptomatic (VAS ≤ 2)	97 (42.2)	49 (45.0)	20 (35.7)	28 (43.1)	
Mild (2 < VAS ≤ 5)	85 (37.0)	38 (34.9)	27 (48.2)	20 (30.8)	
Moderate-severe (VAS > 5)	48 (20.9)	22 (20.2)	9 (16.1)	17 (26.2)	
Allergic rhinitis (SFAR score ≥ 7/16)	54 (23.5)	25 (22.9)	13 (23.2)	16 (24.6)	0.97
Total Mini RQLQ score (0–84)	21.53 ± 16.28	20.88 ± 15.87	20.21 ± 14.46	23.74 ± 18.33	0.42
Activity limitations (0–18)	5.15 ± 4.54	4.86 ± 4.55	4.71 ± 4.12	6.00 ± 4.80	0.20
Regular activities at home (0–6)	1.18 ± 1.59	1.02 ± 1.60	1.05 ± 1.38	1.55 ± 1.69	0.08
Recreational activities (0–6)	1.20 ± 1.63	1.15 ± 1.69	0.96 ± 1.35	1.51 ± 1.72	0.17
Sleep (0–6)	2.77 ± 2.15	2.70 ± 2.16	2.70 ± 2.07	2.94 ± 2.24	0.75
Practical problems (0–12)	2.46 ± 2.88	2.49 ± 2.86	2.43 ± 2.79	2.43 ± 3.03	0.99
Need to rub nose/eyes (0–6)	1.31 ± 1.57	1.44 ± 1.62	1.29 ± 1.53	1.11 ± 1.53	0.40
Need to blow nose repeatedly (0–6)	1.15 ± 1.57	1.05 ± 1.48	1.14 ± 1.57	1.32 ± 1.71	0.53
Nose symptoms (0–18)	3.64 ± 3.92	3.27 ± 3.57	3.43 ± 3.36	4.45 ± 4.80	0.14
Sneezing (0–6)	1.13 ± 1.43	1.04 ± 1.37	1.05 ± 1.35	1.37 ± 1.59	0.30
Stuffy/blocked nose (0–6)	1.62 ± 1.82	1.55 ± 1.82	1.63 ± 1.66	1.74 ± 1.96	0.81
Runny nose (0–6)	0.88 ± 1.38	0.68 ± 1.15	0.75 ± 1.10	1.34 ± 1.80	0.006 *
Eye symptoms (0–18)	3.31 ± 4.08	3.42 ± 4.39	3.09 ± 3.51	3.31 ± 4.05	0.89
Itchy eyes (0–6)	1.00 ± 1.43	1.05 ± 1.62	0.96 ± 1.18	0.94 ± 1.32	0.88
Sore eyes (0–6)	1.07 ± 1.53	1.14 ± 1.68	0.96 ± 1.29	1.05 ± 1.46	0.78
Watery eyes (0–6)	1.24 ± 1.59	1.24 ± 1.65	1.16 ± 1.44	1.32 ± 1.64	0.86
Other symptoms (0–18)	6.96 ± 4.80	6.81 ± 4.78	6.57 ± 4.39	7.55 ± 5.17	0.48
Tiredness/fatigue (0–6)	3.11 ± 1.97	3.15 ± 1.99	3.00 ± 1.87	3.14 ± 2.07	0.89
Thirst (0–6)	1.78 ± 1.76	1.66 ± 1.65	1.68 ± 1.76	2.08 ± 1.92	0.28
Feeling irritable (0–6)	2.09 ± 1.84	2.04 ± 1.86	1.89 ± 1.64	2.34 ± 1.98	0.39

Values are given as mean ± SD or number (%). *: *p* < 0.05; CPAP: continuous positive airway pressure, BMI: body mass index, ODI: oxygen desaturation index, OSA: obstructive sleep apnoea, ESS: Epworth Sleepiness Scale, VAS: visual analogue scale, SFAR: score for allergic rhinitis, RQLQ: Rhinoconjunctivitis Quality of Life Questionnaire.

**Table 2:** Association between baseline characteristics and CPAP usage after three months.

Predictive Factors	Coefficients (Unstandardised)		CI 95%		*p* Value
	B	SE	Lower Bound	Upper Bound	
Runny nose	−0.294	0.139	−0.568	−0.020	0.036 *

*: *p* < 0.05; CPAP: continuous positive airway pressure.

**Table 3:** Changes between baseline and follow-up assessment after 3 months.

Clinical Characteristics	High-CPAP Users (n = 53)		Low-CPAP Users (n = 23)		Non-CPAP Users (n = 27)	
	Paired Difference (FU—Baseline)	*p* Value	Paired Difference (FU—Baseline)	*p* Value	Paired Difference (FU—Baseline)	*p* Value
ESS score	−4.55 ± 4.82	0.000 **	−3.74 ± 5.46	0.006 *	−2.63 ± 5.35	0.017 *
VAS score	−0.08 ± 3.19	0.90	0.43 ± 2.66	0.48	0.81 ± 2.76	0.21
Total Mini RQLQ score	−1.04 ± 17.68	0.96	0.35 ± 11.19	0.69	1.11 ± 12.46	0.83
Activity limitations	−1.08 ± 4.58	0.12	−1.26 ± 4.20	0.28	−0.96 ± 4.38	0.22
Regular activities at home	−0.02 ± 1.66	0.95	−0.52 ± 1.41	0.10	−0.22 ± 1.48	0.47
Recreational activities	0.13 ± 1.80	0.60	−0.04 ± 1.43	0.96	−0.33 ± 1.82	0.31
Sleep	−1.19 ± 2.48	0.001 **	−0.70 ± 2.08	0.11	−0.41 ± 2.31	0.25
Practical problems	0.89 ± 3.74	0.11	0.74 ± 2.53	0.12	0.30 ± 3.00	0.66
Need to rub nose/eyes	0.57 ± 2.14	0.07	0.35 ± 2.14	0.29	0.63 ± 1.62	0.06
Need to blow nose repeatedly	0.32 ± 1.92	0.28	0.39 ± 1.37	0.22	−0.33 ± 2.22	0.42
Nose symptoms	0.60 ± 4.29	0.26	1.22 ± 2.39	0.19	0.52 ± 3.12	0.34
Sneezing	−0.02 ± 1.78	0.66	0.13± 1.01	0.49	0.48 ± 1.25	0.07
Stuffy/blocked nose	0.11 ± 1.90	0.58	0.61 ± 1.85	0.15	0.07 ± 1.73	0.93
Runny nose	0.49 ± 1.59	0.025 *	0.48 ± 1.08	0.056	−0.04 ± 1.60	0.73
Eye symptoms	0.06 ± 5.33	0.86	0.00 ± 3.95	0.94	0.41 ± 4.77	0.84
Itchy eyes	0.17 ± 1.93	0.53	−0.04 ± 1.30	0.86	0.26 ± 1.75	0.69
Sore eyes	0.02 ± 2.13	0.97	0.04 ± 1.69	0.82	−0.15 ± 1.92	0.63
Watery eyes	−0.13 ± 1.93	0.66	0.00 ± 1.48	0.87	0.30 ± 2.03	0.68
Other symptoms	−1.42 ± 5.42	0.042 *	−0.39 ± 5.20	0.30	0.78 ± 3.71	0.46
Tiredness/fatigue	−1.06 ± 2.65	0.005 *	−0.61 ± 2.15	0.16	0.41 ± 2.32	0.32
Thirst	0.15 ± 1.92	0.75	0.78 ± 1.93	0.08	0.48 ± 1.16	0.04 *
Feeling irritable	−0.58 ± 1.85	0.038 *	−0.57 ± 2.11	0.07	−0.04 ± 1.65	0.83

Values are given as mean ± SD. *: *p* < 0.05, **: *p* < 0.005; CPAP: continuous positive airway pressure, ESS: Epworth Sleepiness Scale, VAS: visual analogue scale, RQLQ: Rhinoconjunctivitis Quality of Life Questionnaire.

**Table 4:** Association between change in nasal symptoms and CPAP usage after 3 months.

Predictive Factors	Coefficients (Unstandardised)		CI 95%		*p* Value
	B	SE	Lower Bound	Upper Bound	
Tiredness	−0.262	0.106	−0.473	−0.052	0.015 *

*: *p* < 0.05; CPAP: continuous positive airway pressure.

## Data Availability

The data presented in this study are available on request from the corresponding author. The data are not publicly available due to privacy-related restrictions.
